# Performance indicator as the main and the only goal: a “dark side” of the intervention aims to accelerate HIV treatment entry among people who inject drugs in Kyiv, Ukraine

**DOI:** 10.1186/s12954-019-0279-5

**Published:** 2019-01-28

**Authors:** Alexandra Dmitrieva, Vladimir Stepanov, Ievgeniia-Galyna Lukash, Anna Martynyuk

**Affiliations:** 1 Support, Research and Development Center, Kyiv, Ukraine; 2 Support, Research and Development Center, Kyiv-Mohyla Academy Doctoral School, Kyiv, Ukraine; 3 Convictus Ukraine, Kyiv, Ukraine

## Abstract

**Background:**

To improve healthcare entry and antiretroviral therapy (ART) initiation for HIV-positive people who inject drugs (PWID) in Ukraine, an intervention built upon a successful community-based harm reduction project and the existing best practices was developed. In this article, we present the results of the study conducted in collaboration with one of the recipient organizations of the intervention in Kyiv. The research question was formulated as follows: how does the interaction between different actors work to lead it to a positive outcome (initiation PWIDs into ART) within the limited period of the intervention implementation?

**Methods:**

The central focus of the study was on the work activities of case managers. Their daily routines as well as their interactions with their clients and medical workers were observed and analyzed. Using the institutional ethnography approach, we explore the institutional orders, power imbalances, and social factors that play different roles in coordinating the process of PWIDs entry into healthcare and HIV treatment.

**Results:**

The most intriguing result of the study is that the performance indicator that must be completed in order to receive a full salary—as a way to manage the activities of case managers—produces conditions for them to develop their cooperation with medical workers but leaves the clients and their needs out of this “boat” because interaction with them, in fact, does not help to meet case managers’ goals.

**Conclusions:**

Accountability of case managers’ work assumes the primacy of the result over the process, which makes the process itself less important and the need to achieve the goal becomes the main and the only goal. This can be identified as an unintended consequence of the intervention implementation on the ground, or wider—an unintended consequence of the payment by results practice as a part of the general number-based policy.

## Background

In 2015, the WHO guidelines on antiretroviral therapy (ART) prescription was changed in order to provide immediate access to HIV treatment to all people living with human immunodeficiency virus (PLHIV) regardless of their viral load and CD4 count [1]. In 2016, Kyiv became a Fast-Track City^1^ to consolidate local efforts on combating HIV/AIDS within the global urban framework. The idea of the Fast-Track initiative is complementary to the relevant UNAIDS 90/90/90 targets^2^ and the global approach of “treatment as prevention” which states that adherence to ART treatment reduces viral load and maintains viral suppression, meaning that PLHIV adhering to ART are not a threat to those people who are not infected with HIV [2].

According to recent data of the Center for Public Health of the Ministry of Health of Ukraine [3], there were 127,620 HIV-positive people in Ukraine who were under medical supervision, and 74,780 of them had been receiving ART by the end of 2016. Among the supervised patients, 47, 531 were people who inject drugs (PWIDs), and only 7472 of them were receiving HIV treatment (15.7%), which is disproportionately lower than their share among those in need.

To improve healthcare entry and ART initiation for HIV-positive PWID in Ukraine, an intervention built upon a successful community-based harm reduction project and the existing best practices was developed. The peer-to-peer case management approach which, in modified form, is the core component of the intervention, has been found to be more effective than passive referrals in facilitating linkage to HIV care for various categories of patients, including PWID [4–6].

In this article, we present the results of the study conducted in collaboration with one of the recipient organizations (after—RO) of the intervention in Kyiv. In line with community-based principles, the research question was formulated during a discussion of the most problematic issues in the work of HIV service organizations, between the main researcher (AD) and RO’s program manager. According to RO’s annual report for 2016 [7], from 83 PWID, who were engaged in case management in 2016, only 22 (26.5%) people started ART. We identified the period between case management engagement and ART enrolment as a period of intensive interaction between case managers, medical workers, and clients which may or may not lead to a positive outcome (initiation into ART). Thus, the research question was formulated as follows: how does the interaction between different actors work within the period of the intervention implementation?

It is important to note here that in accordance to the latest data from RO’s report for 2017 [7], of 489 PWID engaged in the intervention in 2017, 253 (51.7%) started ART. These numbers demonstrate an increase in the percentage of people starting ART after engaging in the intervention and at the same time an increase in the number of people engaged in the intervention in 2017 compared to 2016, and also changes in the donor’s performance indicator requirements on the number of people enrolling in the intervention and starting ART. The presented study’s field work started in April 2017 (new program year with new donor’s requirements also started in April) and finished in August 2017. Thus, the researchers became witnesses of how new requirements were “incorporated” into case managers’ everyday working routines and were performed in the context of Kyiv Fast-Track obligations. We found it significant to consider the whole context of interaction as a framework in which interaction between case managers, medical workers, and clients features, and in which the interaction was observed during the study.

## Methods

### Study settings and participants

The main study locations were the following: a community center which also serves as an office; two local confidence rooms where people who have residential registration with the local district can be tested for HIV, make a viral load count and CD4 testing free of charge, and where they can be examined by HIV doctor and get ART; and a City AIDS center—a specialized on HIV treatment clinic where HIV-positive patients with any residential registration can be examined by different doctors at the same building and free of charge. Most of the research time was spent waiting in a line to visit an HIV doctor at the local confidence rooms and City AIDS (in a line to visit HIV doctors and other specialized doctors) Center and getting around between these locations.

The shadowing was conducted with 4 case managers, 1 male and 3 female ones. Only 1 case manager had had lengthy experience of social work—around 10 years, 2 years of which he worked as a case manager. The 3 female case managers had had experience as social workers in other organizations (no more than 1 year), and all 4 case managers had had a 2-year experience of working as case managers. None of the case managers had a degree in social work, public health, or medical care. All of them, however, had taken part in a number of specialized certified trainings in social work and case management. All of the case managers had previous personal experience of drug use, with different durations of non-use remission (from 3 to 10 years). One of the case managers is a patient of an OST program.

When the shadowing period was completed, two focus groups with medical workers and case managers were conducted. The medical workers were represented by two HIV doctors, four nurses, and two psychologists. The four shadowed case managers participated in the focus group meetings as well. In total, each group included seven participants, excluding one moderator and two researchers taking field notes during the group meetings. The confidence rooms in two city districts were used as the venue for the focus group meetings. The duration of each focus group was around 1 h and 30 min.

### Data collection and analysis

The study conducted institutional ethnography (IE) using a thick description technique. IE is a method for studying the social interactions that shape and organize everyday life experiences [8–11]. The focus in IE is on the ongoing structural and social determinants of everyday activity [12]. These determinants include institutional work processes—the implicit norms and explicit rules that organize daily work. As McCoy states, “the health care of a person living with HIV involves a complex, daily work process that loops from the home and everyday spaces of the individual into the sites of professional medical service and back again” [13]. We may add to McCoy’s statement that within the framework of our research, in the chain of a person, their environment, and the space of medical institutions, another fully-fledged actor appears. The case manager is involved in the contexts of both social work and the global approach to counteracting the HIV epidemics.

The central focus of the study was on the work activities of case managers. Their daily routines as well as their interactions with their clients and medical workers were observed and analyzed. Using the IE approach, we explored the institutional orders, power imbalances, and social factors that play different roles in coordinating the process of entry into healthcare and HIV treatment. The goal of the IE in this study was to observe and to specify how the pre-determined orders and algorithms of initiating PLHIV into HIV treatment work in the case of PWID clients/patients.

The “shadowing” technique was used as the main data collection method. Shadowing offers access to “invisible” aspects of work and social life organization [14]. “Participant” shadowing suggests asking questions or gaining an insight at the moment which is significant [15], particularly when the work being done is difficult to observe [16]. Following McDonald’s advice—“never go [shadowing] in cold” [16], both field researchers were familiar with RO’s work. One of the researchers (EL) had previous experience of outreach work with female sex workers at RO, another researcher (AD), serving as an ethnographic field researcher, spent several weeks observing RO’s clients and social workers’ activities at RO’s community center. Two researchers, working at the same time but observing different case managers, carried out all the shadowing sessions. Each day the researchers changed places to create the most objective picture of the observation. Three days of observation were conducted with each of the four case managers working at RO for a total of 12 shadowing sessions. There were breaks between the observations due to a number of official holidays within the fieldwork period.

At the end of each day of shadowing, both researchers filled in an online field diary in Google Docs to make the process of data collection easier and more manageable (by the end, the overall volume of the field notes was more than 19,000 words). The focus group discussions were transcribed verbatim. The transcripts and the shadowing field notes were included into the trial version of Atlas.ti software for qualitative data analysis for further work with coding and categorizing.

A grounded theory approach was used for the data analysis [17]. An initial list of broad categories for coding was developed prior to the shadowing, and the focus groups were based on researchers’ previous research experience. The field notes were also structured according to a pre-defined scheme. We theoretically identified the main types of interactions that occur in the process of case management: (a) interactions with different people and the surroundings, and (b) activities which case managers produce when they do not interact with other people but perform their routine paperwork.

After primary analysis of the shadowing field notes, four separate topics were formulated for discussion during the focus groups: case managers and medical workers as part of one effective system aimed at facilitating entry to HIV care and ART initiation; barriers to HIV care and ART initiation; and ways and techniques to facilitate PWIDs’ entry to healthcare, ART initiation, and adherence to ART. Both focus groups were moderated by one researcher (AD). Two other researchers (EL and AM) took field notes during the focus groups (the third researcher (AM) joined field work at the stage of conducting the focus groups). When all the focus groups’ audio records had been transcribed and coded (the shadowing field notes had been coded before), each category was assigned a set of corresponding codes. The process was accompanied by extensive theoretical memo writing, which is a key component of grounded theory analysis [17, 18].

## Results

### A context of case management implementation

#### A short description of case managers working algorithms

Case managers engaged in the intervention operate within previously developed working algorithms, performance indicators (number of people engaged in the intervention, number of people registered at AIDS centers, number of people who have started ART), and time frames for case implementation defined by the donor.

Interaction between case managers and their clients usually starts from the client’s identification as a person living with HIV by a case finder or a social worker whose duty is to provide rapid HIV testing facilities for PWID. HIV testing is awarded by a small cash incentive, as well as recruitment of other PWID for HIV testing if a person was tested positive. Then, the case finder or the social worker introduces the newly identified client to а case manager. After a conversation with the potential client, the case manager suggests that they engage in case management to assist them in a process of engagement in HIV care and treatment. If a person confirms their participation, a case manager signs an informed consent form and a plan of assistance and suggests that a client sign it as well. After a receipt of all the signed documents, a case can be considered “opened.” A case is considered to be “closed” 1 month after a client has been initiated into ART; in practice, that means that a case manager meets a client for the last time 1 month after ART initiation to assist them in undertaking ART on their own for the first time. As motivation for a client’s first independently taken ART, the case manager gives them a small cash incentive.

The maximum duration of each case is 5 months. If within a 2-month-period the client is not registered at the AIDS Center, the case is recommended for closure, or to be negotiated with the corresponding donor’s specialists concerning prolongation of the first stage for an additional month (such a request should be supported by an evidence-based explanation in written form from the intervention program manager). According to the working plan for October 2017, each case manager had to start 8 new cases per month. The indicator for the number of people initiated into ART is 6 people initiated in ART per month per one case manager. If the ART indicator is not achieved by the end of a month, the case manager does not receive the full amount of their monthly wage. But, if a case manager has initiated 5 people into ART and can reasonably prove (and provide specific papers) why the 6th person has not been initiated by the end of that month, the case manager will get their full monthly wage.

Every day upon returning to the community center, case managers update the information on the clients they lead (each of them has a special notebook where they have to write up plans of action for each case and make notes of what is already being done), make phone calls, and arrange the next meetings. Each action taken in respect of a client must be documented, both by the case manager and at a corresponding medical institution by putting signs and stamps in “the coupon” that serves as a reporting document for program managers at RO and for the donor as well.

### Need for “overtravelling” as a result of a lack of integrity in the services

While shadowing the case managers’ daily routines, we were surprised by all the traveling done by the case managers. Each case manager has several clients living and registered (sometimes at the same time) in different city districts. To be engaged in HIV care and to start ART, any patient should use local (where they are registered) district facilities—local confidence rooms (where a patient could be tested for HIV and get ART) and local regular clinics (where a client could be examined by necessary doctors to be prescribed suitable ART regimen at a local confidence room), or City AIDS Center where a patient with any residential registration can be registered examined by all necessary doctors to get HIV care and treatment.

In practice, in most cases, because of several reasons which will be described later, the case managers prefer to avoid local regular clinics and turn directly to the City AIDS Center.

The City AIDS Center is located at a 20-km distance from the community center and the local confidence room next to it. Traveling to the City AIDS Center is long and complicated enough, because of an absence of a subway line nearby. Getting by subway to the City AIDS Center’s subway station takes around 40 min. Thus, the whole trip takes around 1 h and 30 min (it depends on the time of the day when the trip starts and a traffic). The second observed local confidence room does not provide case managers with more optimized opportunities for time and travel management. The only difference is that the nearest subway station is not as far as from the first confidence room.

Due to the lack of integrity within services, to make the clients start their ART, case managers have to move between several remote locations in each separate case or to centralize their activities within the City AIDS Center. The second option optimizes the way case management routines are organized, but do not consider the clients’ needs and location.

### PWID as “difficult clients/patients”

Considering the time spent on travel, another time-consuming aspect of case managers’ daily work is their need to wait for their clients: clients are often late or do not come to meetings at all, do not answer the phone calls, or even suddenly die.

*- Hello, may I talk to Valera?*

*- Valera is dead (his girlfriend answered)*.

*- Ahhh…Valera is dead, but will you come to us?*

*Kolia (case manager) called the client whom they tested 8 days prior, his girlfriend answered and it turned out that the client had died. Kolia paused for a second, but almost immediately asked whether the girlfriend was going to undergo treatment, the girl agreed to call him on Monday and come to the confidence room on Tuesday, and only after that Kolia offered his condolences (shadowing field notes)*.

Such delays and disappearance could be explained by the nature of drug-dependent behavior and the clients’ reluctance for the next steps after engaging in case management. Concerned that they would lose freshly identified HIV-positive client, case managers often try to accelerate the process of signing the documents necessary to open the case. However, newly registered as case clients often are not formally prepared for engaging in HIV care (i.e., registration for medical supervision) because of things which are typical among PWID but make them different from “normal” people. First of all, they have to deal with drug dependence, i.e., to find money, to buy, and to use drugs before any meetings with case managers or doctors, or other people. Then, they have to overcome bureaucratic barriers like missing passport, absence of local residence registration, or other documents necessary to be engaged in HIV care, to find money for travel between their home, confidence rooms, AIDS Center, and other places.

*The client did not come for the second time, although, according to Valia (case manager) and Dima (social worker), he had been motivated to undergo treatment until it turned out that he needed a passport to be registered at the AIDS center. Then it turned out that he moved from another city, but was living with his girlfriend in Kyiv, so Valia wrote and sent a statement giving permission for the client to be registered at the Kyiv AIDS center by the district passport office. The client “went” to receive this permission, then he “disappeared off the radar” (shadowing field notes)*.

As we mentioned before, there are several reasons why clients and case managers prefer to make the long trek to the City AIDS Center instead of seeking medical help at their local clinics. One of those reasons is stigma and discrimination which PLHIV and PWID may meet with at regular clinics. Typically, a doctor from a regular clinic, who does not benefit in any way from working with patients from the key populations, does not have specialized knowledge and practice (or does not want to have them; we assume that, since the beginning of HIV epidemics in Ukraine, most of the medical staff had to be additionally trained). In such cases, it is very difficult for a case manager to cushion the blow of such an attitude towards their client.

*A separate topic are the “other” doctors. We come to a radiologist, an old woman, to make an X-ray, she shushes us as though we are dogs: “In half an hour” and slams the door, we go out. Katya says that she [the doctor] behaves the same way with clients and they have already complained about it (shadowing field notes)*.

*One day I got sick and called a doctor to visit me, two elderly women arrived, one of whom was palpating my stomach, the other was asking questions about my health. I reported that I was HIV +. The one that was palpating my stomach abruptly pulled away and ran to wash her hands, the second one started yelling that it was crazy not to warn in advance about my HIV status! (a story told by an HIV positive social worker, shadowing field notes)*

A lack of visible progress in the acceptance of PLHIV and patients from key populations by regular medical workers results in a lack of confidence and negative attitudes towards the medical workers in general, and self-stigmatization among PWID and other key groups. Even medical workers working in the confidence rooms reported that being part of an HIV service, they become stigmatized as well—by their colleagues from the health care system, by other patients, by their friends and other people:

*Sometimes people open a doorknob in my office using napkins (psychologist-1, focus group)*.

### Achieving the performance indicator as case managers’ main goal

There is no obligatory activity related to the clients’ needs assessment and assistance according to identified needs in the case managers’ working algorithms. The idea of case management here is that if a client is HIV+, a client’s need is HIV treatment. Thus, a mimicry appears of the working obligations of case managers to their clients’ needs, i.e., undertaking ART. However, it does not change the fact that clients may have other plans and needs, from their point of view, even more life-saving than ART. One of such needs is the clients need for money, even a small cash incentive may serve as a way to attract PWIDs to make an HIV test or even to visit a doctor and to start ART but does not guarantee their retainment in treatment:

*Then we suddenly run to the confidence room because Katia’s old client has come. It turned out that a year ago, when he had engaged in ART with Katia’s help, he did not start taking it. Now, he has come because the other case manager (Kolia) called him and promised 70 hryvnas if he comes and undertakes HIV testing. Accidentally, the client and Katya meet, Katya is shocked, she grabs him and we run to the confidence room (later she describes it literally as “I grabbed his hair and we ran”). All the way back Kaiya is resentful again and keeps asking him [the client] how he could come for testing to Kolia instead of calling her. As a result, the client cannot stand it and says: “You can keep “sawing me” as you wish to, just agree between you two, I’m not interested in taking part in this”. Katia's behavior is like the behavior of an animal returning its kids running away from the den. After visiting the confidence room, we go back to the community center. During the breaks between phone calls and paperwork, Katia is still crazy about Kolia’s (the case manager’s) behavior. She says that she will go to her boss and tell her the story of how Kolia has stolen her indicator. Kolia is nervous as well, he is almost crying (shadowing field notes)*.

To complete their work and to achieve their performance indicators, the case managers use different strategies to interact with their clients. The prevailing strategy is when a case manager takes a complete responsibility of how the clients act during the case—deciding for them who will speak with a doctor or who will wait in line. From four case managers we were shadowing, three case managers were implementing this strategy in a such way:

*Katia solves any question with the doctor instead of the clients asking them to wait outside the door (shadowing field notes)*.

The perception and description of how similar situations are managed can differ from case manager to case manager, depending on their individual characteristics and personal experience. But one thing remains the same—the complete power of a case manager over the situation. One of the four shadowed case managers implements another strategy which seems to be more time-consuming and may deviate from the working algorithm but could be described as more “empowerment-oriented” as it aims to develop the client’s own decision-making and responsibility over the situation. Unlike the first strategy, this one aims to provide emotional support to the client for building a long-term relationship and to promote adherence to a certain type of relationship aimed at mutually beneficial cooperation and the client’s own ability to cope with different types of situations.

*On the way from the community center to the confidence room, Kolia told me how his understanding of case management changed from the moment he began to work as a case manager. When starting, he thought that when coming to a medical institution with a client, the line of people seeing them should part, letting them go ahead. Faced with the fact that it did not happen, Kolia realized that the people in the line do not owe him or his client anything, and it was okay to wait for their turn. As Kolia explains, he was worried that when the client saw the line, they would feel cheated, because Kolia had promised that there wouldn’t be any waiting. In practice, after some cases, it became clear that waiting in line is just normal, and it is possible to explain this to a client (shadowing field notes)*.

However, this strategy is far from always being successful, because the time spent on its implementation fits poorly into the working performance indicator plan. Failure to achieve a certain number of actions on the part of the client at the specified time leads to shifting attention to new cases to complete the case manager’s working duties. Discussing these strategies with case managers, we concluded that such a “soft” strategy is not supported by the other three case managers because it does not help to achieve performance indicators and, as they claim, does not work well with PWIDs.

After the case is closed, the case manager refers the client to the project for treatment adherence support funded and managed by another donor and organization. Similarly to the case managers, those social workers provide their services under the system of performance indicators achievement as well:

*While we were at the AIDS Center, Valia met a guy with whom she had been in rehabilitation, now he works at the project for treatment adherence support. He asked her if they could have a talk; then it turned out that he had asked her to hand over the clients to him personally because he had to achieve his “indicator” (shadowing field notes)*.

Thus, to get salary, case managers have to develop the most optimized way to achieve results according to their obligation to implement a monthly performance indicator plan. Such accountability of case manager’s work assumes the primacy of the result over the process. Nevertheless, to achieve the goal, case managers have to identify strategies facilitating their efforts.

### Facilitators for achieving the goal

#### HIV doctors performing their duties in “the shoes” similar to case managers’

The same with case managers, medical workers (HIV doctors, in particular) have a monthly performance indicator plan determined by Kyiv’s participation in the Fast-Track initiative. The activities of case managers are framed by two main work results and performance indicators—the number of PWID who engage in HIV care and the number of PWID who start ART; thus, their responsibility and working obligations are considered to be completed at the level of ART initiation and cover only PWID. The responsibility and working obligations of HIV doctors and medical workers working at confidence rooms and the City AIDS Center cover any HIV-positive people who are registered for medical supervision and engaged in HIV care, and their performance indicators include the number of people with a zero viral load that could be suppressed if only a patient is adhering to ART. As a result, HIV doctors’ work load as well as their performance indicator increase tremendously.

*There are a lot of people at the HIV-doctor’s office - the doctor herself, a nurse, two social workers from the Care and Support program, one client who has come to take part in the survey, our client, Kolia (case manager) and I, and all these people are in the space of only 15 square meters. Because of the quantity of people, it becomes noticeable that the functional sense of medical practice is shifted very much towards paperwork and attempts to optimize the workflow with the help of computers (there are 4 computers at the doctor’s office), and there is still an endless number of papers lying all over the place. The interaction between the client and the doctor looks like a business meeting – short and concrete (shadowing field notes)*.

Cooperation with case managers as a way to decrease doctors load with “difficult” patients has become a “win-win” situation. Working at optimizing the time spent on each patient, HIV doctors warmly welcome the way of interaction with PWIDs suggested by case managers. Thus, interaction between doctor and patient replaces by a duo of a case manager and an HIV doctor. Case managers take over actions that clients could do themselves: they arrive ahead of time to wait in line for the doctor; they communicate with doctors instead of a client; they pick up ART from a confidence room upon the client’s request; they make appointments with a doctor instead of the client, or they create opportunities to take lab test results without waiting in line, etc. No one really thinks about whether there is a reasonable need for such actions, they just do it because it is faster and easier than to wait until the clients do it themselves.

*Oh God, how these girls (case managers) cherish and lead him [a client] to the registration at the AIDS center … They get him coffee, water, have him do the X-ray, then take him back, get him cigarettes. Well, very good! (nurse 2, focus group)*.

*Last night we agreed to meet with a case manager and a client who did not fit the ART scheme in the morning. When I met the case manager, it turned out that we had met beforehand to wait in line to see a doctor, one and a half hours before the doctor started her shift, and the client would arrive at 11.30 at the same time as the doctor (shadowing field notes)*.

### Case managers “with privileges”

Spending lots of time in the confidence rooms and the City AIDS center, case managers make attempts to develop a more informal relationship with medical workers rather than a formal, professional one, justifying it as a way to increase the number of privileges for their clients. This also increases the number of privileges available for case managers themselves. One of the examples of such privileges is to have an access to “the doctor’s journal of patients who failed to follow up” (patients who failed to follow up at the confidence room or the AIDS Center and have never been clients of the case managers before). For case managers, to get the “lost patients” from doctors directly means to skip the standard process of case management—they do not need to register such cases at the AIDS Center as they all have already been registered there and do not need to visit all the other necessary doctors. To get such an opportunity, this “case manager with privileges” is ready to even do strange and humiliating things. One such example is to do manicures and pedicures for the HIV doctor.

*When we go outside, Anna explains to me whispering, that she works closely with an HIV-doctor and the client who has just appeared is not a “typical” client, his appearance is not just merely a result of a standard scheme when a case finder or a social worker tested him positive. He has come directly from a doctor. There are patients who failed to follow up for medical supervision, and to re-engage them in care and treatment the HIV-doctor may refer such patients to a case manager, not vice-versa as it normally happens (shadowing field notes)*.

Shadowing other case managers, we found out more conventional variations of attempts by case managers to achieve more informal relationships with medical workers and get some privileges as a result: one of the case managers collects pens for some time and then passes them on to radiologists so that they would let his clients be served without waiting in line, another case manager gives notebooks and paper sheets as presents to doctors, and another one presents spring flowers and says compliments to doctors. It is important to outline that case managers themselves do not see these actions as gifts or bribery, referring to it as an expression of an attentive attitude towards doctors. Such signs of informal attentive attitude strengthen the informal side of the relationship between case managers and medical workers and probably bring more “humanity” into their relationships. However, it produces and normalizes cooperation between case managers and medical workers but does not support in any way the development of a relationship between the clients as future patients of ART and their doctors.

## Discussion

We assume that considering a positive outcome of intervention aimed at accelerating HIV treatment among PWID as a number of PWID initiated into ART is not productive without considering the number of PWID adhering to HIV treatment and having their viral load suppressed. Unfortunately, we have not found any open data or statistics regarding the number of PWID adhering to ART in Ukraine, as well as any qualitative studies that would explore the whole process—starting from HIV care engagement to promoting ART adherence among PWID in Ukraine. It is also a limitation of our study because the intervention finishes at the level of ART initiation and does not monitor and evaluate the outcomes of the intervention ahead of the number of people who are taking part in the intervention and start taking ART. According to literature on performance-based financing, implementation of performance-based payment policy may reinforce certain forms of “wrong” behavior such as “gaming,” i.e., activity that facilitates the attainment of the targets without contributing to a real or intended improvement in health outcomes [19–21]. The existing system of the intervention results evaluation also contribute to reproduction of such behavior by considering short-term results of case management only, or in other words, by using the “cherry picking” strategy to evaluate and present incomplete picture [21, 22].

There is a sufficient number of studies regarding barriers to HIV care engagement and treatment among PWID in Ukraine. Most of them describe effective integration of health services as a way to improve multiple healthcare outcomes among PWID, including retention in HIV care and TB treatment [23–26]. According to the results of our study, case managers, most of whom are peers and represent the group of “former” PWID, do not provide any specific consultations or actions to improve their clients’ access to OST, as this activity is not a part of the case managers’ working algorithm. At the same time, many disparities of case managers’ work related to the fact that their clients are drug dependent, which may influence their motivation to engage in HIV care—or at the level of everyday life interactions—to come on time for meetings with the case manager, to answer phone calls, to be able to wait in line to see a doctor, to communicate adequately, etc. S. McGill, in her PhD work on the impact of the Global Fund programs on HIV prevention policy and services in Ukraine in 2003–2012, declared that “the nomenclature of prevention services and the number of people to be covered were already pre-determined, and NGOs could neither define the needs for prevention services, nor choose services themselves” [27]. Confirming McGill’s thesis, we can add that the nomenclature of treatment services provided by NGOs in Ukraine is also pre-determined by the frames of a concrete intervention developed by the donor. This phenomena of pre-determined results and frames of activity can be interpreted as some of the barriers to healthcare integrity as it aimed at achieving a goal within pre-determined frames and reproduce rigidity of post-soviet systems but already on the level of NGOs [27, 28].

Another common barrier which appears probably in every study focusing on people who use drugs, either HIV-positive or not, is stigmatization and discrimination at any level—structural, group or inter- and intra-personal [29–32]. The reality of suspicion, mistreatment, and hesitation from healthcare providers treating PWID, and particularly HIV-positive PWID, has been well documented in different regions of Ukraine [25, 33, 34]. This study confirms the results of previous stigma-focused studies and also broadens the understanding of stigma consequences through observing case manager’s activities aimed at avoiding regular clinics. One of the reasons why centralization of case management activities at the City AIDS Center happens is the traumatic experience of previous clients and case managers themselves who met with stigmatizing and humiliating attitudes in regular clinics. In addition, avoiding regular clinics could be explained by case managers’ conscious choice that saves their time and represents an easier way to reach the targets. It could be suggested that every case manager’s new client will not try to go there because the case manager will advise them to go to the City AIDS Center. Because of that, the level of existing stigma will hardly ever change in this environment and the integrity of health services overall will not reach the level of services at the only one AIDS Center in a three-million population city where medical workers are prepared to meet and treat people from key groups.

In this study, we documented case managers’ strategies to strengthen the informal side of their relationship with medical workers as a way to increase the number of privileges which help case managers in achieving their goals. By “the privileges,” we understand concrete results of establishing informal relationship between case managers and medical workers such as decreasing time spent in a line to a doctor, getting access to the doctor’s journal of patients who failed to follow up, etc. As Oxman and Fretheim state “the more remote the point of service delivery, or the more complex the service to be delivered, the more likely it appears that contracts or agreements will be governed by informal means” [35]. A comprehensive collection of informal practices including informal practices related to medical care is presented in *The Global Encyclopedia of Informality* edited by A. Ledeneva [36]. The described case managers’ strategy was widely known in the Soviet Union countries as “blat contacts” (personal networks) which “were commonly used to obtain goods and services in short supply or to circumvent formal procedures” [36]. Thus, a common practice of the Soviet Union period is reproduced by the case managers living and working in the post-soviet era and experienced the anti-corruption Maidan movement in the not such remote past (in 2014).

The lack of healthcare service integrity and a high level of stigma were identified as barriers to HIV care a long time before this study was planned. Yet, the most intriguing result of our study is that we may have potentially identified another barrier which has not been described before as a barrier in Ukraine, but was identified as such in other countries where global donor agencies operate [35]. The performance indicator must be completed in order to receive a full salary—as a way to manage the activities in the field of efforts to accelerate HIV treatment coverage. It produces conditions for developing cooperation between case managers and medical workers but leaves the clients out of this “boat” because interaction with clients, in fact, does not seem to help meet case managers’ goals. Thus, performance numbers took priority over providing services to the clients/patients [37]. Owczarzak and her colleagues explore the phenomenon of an “audit culture” represented through evidence-based interventions implementation, monitoring, and evaluation in the USA since 2011 [37–41]. Examining how the pre-packed interventions are implemented by community-based organizations, Owczarzak explores how number-based reporting and accountability practices affect the ways in which service providers interact with clients, their roles within organizations, and the work context more broadly. Our study also conducted an analysis of interaction between PWID, case managers, and medical workers operating within the frames of standardized intervention managed by number-based outcomes, but we were limited by the possibility to only study one organization out of the 35 organizations implementing the intervention in Ukraine.

## Conclusions

The lack of data regarding the PWID retainment in HIV treatment in Ukraine creates difficulties in answering the question on how the interaction among PWIDs, case managers, and medical workers within the frames of intervention aims at accelerating engagement into ART actually works. We can identify the number of people engaged, but there is no information on the number of PWIDs retained in HIV treatment. Thus, only short-term results pre-determined by a donor can be demonstrated, but not long-term results which are identified as a final element (adherence to ART = zero viral load) of the 90/90/90 strategy.

However, the presented study enabled us to explore the period of intensive interaction between case managers, medical workers, and clients which may or may not lead to a positive outcome (initiation into ART). Operating within the pre-determined case management algorithm based on performance indicator plan, the case managers develop, in fact, detrimental strategies which can be successfully incorporated in existing structure of the healthcare system to achieve their pre-determined goals in the limited period of time. Despite many previous attempts to reform the soviet-type social order and to struggle post-soviet corrupted practice, the most optimized way to achieve case managers’ goals is to establish informal, personal relationship with medical workers to increase the number of privileges in getting access to opportunities that make their work more predictable and successful in terms of performance indicator implementation. In other words, to achieve their goals, the case managers have to reproduce a low-level corruption to accelerate ART initiation among PWID clients. Thus, meeting case managers’ or donors’ targets do not reflect any progress in overall health system development that is particularly crucial in the times of transition from the Global Fund to the Ukrainian Government funding.

Such accountability of case managers’ work assumes the primacy of the result over the process, which makes the process itself less important and the need to achieve the goal becomes the main and the only goal. This can be identified as an unintended consequence of the intervention implementation on the ground, or wider—an unintended consequence of the payment by results as a part of the general number-based policy. We assume that further research developing the idea of unintended consequences of the number-based policy will be very productive to improve understanding on the long-term results of implementing standardized interventions aiming at combating HIV in Ukraine in the frames of 90/90/90 strategy.

## Abbreviations

AIDS: Acquired immunodeficiency syndrome
ART: Antiretroviral therapy
CDC: Centers for Disease Control and Prevention
HIV: Human immunodeficiency virus
IE: Institutional ethnography
OST: Opioid substitution therapy
PLHIV: People living with HIV
PWID: People Who Inject Drugs
TB: Tuberculosis

## Footnotes

1 The Fast-Track Cities is a global partnership between the City of Paris, International Association of Providers of AIDS Care (IAPAC), the Joint United Nations Programme on HIV/AIDS (UNAIDS), and the United Nations Human Settlements Programme (UN-Habitat) in collaboration with local, national, regional, and international partners and stakeholders.

2 90% of people living with HIV know their HIV status; 90% of people living with HIV who know their HIV status are accessing ART; and 90% of people living with HIV receiving treatment achieve viral suppression.
